# Enhancing the Effectiveness of Consumer-Focused Health Information Technology Systems Through eHealth Literacy: A Framework for Understanding Users' Needs

**DOI:** 10.2196/humanfactors.3696

**Published:** 2015-05-20

**Authors:** Lars Kayser, Andre Kushniruk, Richard H Osborne, Ole Norgaard, Paul Turner

**Affiliations:** ^1^ Department of Public Health, University of Copenhagen Copenhagen Denmark; ^2^ School of Health Information Science University of Victoria Victoria, BC Canada; ^3^ Deakin Population Health Strategic Research Centre, Deakin University Melbourne Australia; ^4^ eHealth Services Research Group, University of Tasmania Hobart Australia

**Keywords:** eHealth literacy, requirements, user involvement

## Abstract

**Background:**

eHealth systems and applications are increasingly focused on supporting consumers to directly engage with and use health care services. Involving end users in the design of these systems is critical to ensure a generation of usable and effective eHealth products and systems. Often the end users engaged for these participatory design processes are not actual representatives of the general population, and developers may have limited understanding about how well they might represent the full range of intended users of the eHealth products. As a consequence, resulting information technology (IT) designs may not accommodate the needs, skills, cognitive capacities, and/or contexts of use of the intended broader population of health consumers. This may result in challenges for consumers who use the health IT systems, and could lead to limitations in adoption if the diversity of user attributes has not been adequately considered by health IT designers.

**Objective:**

The objective of this paper is to propose how users’ needs and competences can be taken into account when designing new information and communications technology solutions in health care by expanding the user-task-context matrix model with the domains of a new concept of eHealth literacy.

**Methods:**

This approach expands an existing method for supporting health IT system development, which advocates use of a three-dimensional user-task-context matrix to comprehensively identify the users of health IT systems, and what their needs and requirements are under differing contexts of use. The extension of this model involved including knowledge about users’ competences within the seven domains of eHealth literacy, which had been identified based on systematic engagement with computer scientists, academics, health professionals, and patients recruited from various patient organizations and primary care. A concept map was constructed based on a structured brainstorm procedure, card sorting, and computational analysis.

**Results:**

The new eHealth literacy concept (based on 7 domains) was incorporated as a key factor in expanding the user-task-context matrix to describe and qualify user requirements and understanding related to eHealth literacy. This resulted in an expanded framework and a five-step process, which can support health IT designers in understanding and more accurately addressing end-users’ needs, capabilities, and contexts to improve effectiveness and broader applicability of consumer-focused health IT systems. It is anticipated that the framework will also be useful for policy makers involved in the planning, procuring, and funding of eHealth infrastructure, applications, and services.

**Conclusions:**

Developing effective eHealth products requires complete understanding of the end-users’ needs from multiple perspectives. In this paper, we have proposed and detailed a framework for modeling users’ needs for designing eHealth systems that merges prior work in development of a user-task-context matrix with the emerging area of eHealth literacy. This framework is intended to be used to guide design of eHealth technologies and to make requirements explicitly related to eHealth literacy, enabling a generation of well-targeted, fit-for-purpose, equitable, and effective products and systems.

## Introduction

A major factor identified as a point of failure in the development and implementation of health information systems is limited understanding of users, their needs, and the contexts in which the systems are used. Measurable benefits from involving users in the design and development of information technology (IT) have been demonstrated in some studies [1]. However, the comparison of outcomes across these studies is often problematic due to the different ways users are defined, engaged, and the degree to which they are involved in real-world settings. Importantly, this has tended to obscure the way in which different users’ needs, capabilities, and contexts have an impact on the safety and usability of eHealth systems. Because eHealth systems increasingly focus on enabling autonomous use by health consumers, the implications of these differences for patient safety require even closer attention and investigation.

For health consumers, access to networked information technologies has primarily emerged as a way of either complementing or by-passing conventional sources of health-related information. These technologies are increasingly being used to share health information, personal experiences, and knowledge of medications and medical services, as well as to offer support and the tracking of personal health care [2]. The capacity of health consumers to successfully use these systems links back to the classical problem of communication and challenges arising from any misalignment in the communication triangle (the sender, the message, and the recipient) [3]. When health IT is used for information and/or communication (the message), outcomes rely heavily on an alignment of the system’s (the sender’s) capability to address and acknowledge the health consumer’s (the recipient’s) attributes. From the user perspective, the consumer needs to possess both the confidence and skills to acquire information, understand it, and actively appraise it [4]. When using digital media, this means that the consumer needs to be *eHealth literate* [5].

From a health IT design perspective, to ensure that systems have a high level of usability (ie, high efficiency, effectiveness, and generate satisfaction for users) [6], it is necessary that the needs, capabilities, and contexts of use of these users are known and incorporated into the IT design. For more than a decade, different approaches have been taken to illuminate the users’ interaction with IT systems (eg, by assessing their digital literacy or confidence in these systems) [7-9]. In 2006, Norman and Skinner [10] proposed a model of eHealth literacy, taking six core literacies (ie, traditional, media, computer, information, science, and health literacy) into one concept, defined as “an individual’s ability to search for, successfully access, comprehend, and appraise desired health information from electronic sources and to then use such information to attempt to address a particular health problem”. They also developed the eHealth Literacy Scale (eHEALS) instrument [10] that can be used to measure eHealth literacy. However, transformation of health IT from simple and static websites (Web 1.0) to more complex and dynamic applications, online services, and social media (Web 2.0) has superseded the original conceptual base of the eHEALS and other instruments [11].

As private sector providers have entered the social media market to deliver support to patients online [12], other trends highlight the negative impacts of eHealth arising from misinformation [13] and/or reinforcement of negative behaviors [14], ultimately compromising the consumers’ safety.

This calls for new ways for providers to address their consumers being aware of their needs and capabilities. In response to these challenges, we have developed a new concept of eHealth literacy to be reported in detail in due course.

This paper presents a framework grounded in a multidisciplinary approach that supports the modeling of user requirements and understanding of their needs by integrating a new concept of eHealth literacy with an expanded user-context-task matrix advocated for safe eHealth systems design.

The framework presented is structured as a five-step process aimed at supporting eHealth system designers to improve their understanding and more accurately address end-users’ needs, capabilities, and contexts of use to improve the effectiveness and broader applicability of consumer-focused eHealth systems and applications. Using this framework during design will make requirements related to eHealth literacy explicit and contribute to the delivery of better-targeted, fit-for-purpose, equitable, and safe eHealth applications and systems to maximize consumer empowerment and health outcomes while reducing health inequalities. It is also anticipated that the framework will raise awareness of eHealth literacy issues among policymakers involved in the planning, procuring, and funding of eHealth infrastructure, applications, and services.

## Methods

### eHealth Literacy Concept Development

Although the focus of this paper is on presenting a framework for understanding users’ needs to enhance eHealth systems design, it is useful to briefly describe the fieldwork approach that led to the development of the new concept of eHealth literacy used within this paper.

The key points of the new “eHealth literacy” concept are based on a highly structured and rigorous approach to questionnaire development—the validity-driven approach [15]. This is the same methodology that was used in the development of the Health Literacy Questionnaire [16] and in numerous other widely used questionnaires [17-21].

Between June and August 2012, eight consultation sessions were conducted by two of the authors of this paper (ON and RHO) with participation of computer scientists, academics, health professionals, and patients recruited from patient organizations and primary care. During each session, a concept map was constructed based on a structured brainstorm procedure, statement generation, card sorting, and computational analysis [22]. A total of 458 statements were grouped into 68 clusters initially using hierarchal cluster analysis based on the participant sorting data undertaken in each session. Finally, qualitative synthesis was applied to reduce the clusters to smallest number of conceptually distinct concepts of eHealth literacy (Textbox 1). The results were confirmed by an Internet-based consultation with stakeholders.

The resulting seven domains were further grouped and reflected three overarching themes related to end users and technology. The first theme (capabilities) was the end-users’ capabilities within the areas of health, information, and technology; the second theme (access to technologies) was the end-users’ relationship to, or perceptions relating to, interacting with technology—more specifically, whether technology was perceived as being accessible and suits individual needs; and the third theme (experience using technologies) was the experience of benefits of using technology including being in control.

The concept is not only a template for an ongoing development of a new eHealth literacy instrument but also a model grounded in the experiences of modern-day IT users.

As a result, the seven domains of this eHealth literacy concept could be integrated with conventional IT design processes to enhance the understanding of users’ needs among designers of eHealth systems and applications. The next section of the paper focuses on the integration of the new eHealth literacy concept (encapsulated in the seven domains described in Textbox 1) with the expanded user-task-context matrix.

**A new concept of eHealth literacy.**

1. Capabilities
  Knowledge about one's own health (Domain 1)
  Know about the body’s basic functions and structure and own current health status. Aware of risk factors and how to avoid them or reduce their influence on own health.
  Ability to interact with information (Domain 2)
  Able to read, write and remember, apply basic numerical concepts, and understand context-specific language (e.g., health, IT or the user’s native language, as well as critically appraise information. Know when, how and what information to use.
  Ability to engage with technology (Domain 3)
  Being comfortable using computers and other digital media for handling information.
2. Access to technologies
  Access to technologies that work (Domain 4)
  Have access to technologies (e.g. computers and other digital media) that the users trust to be working *when* they need it and *as* they expect it to work.
  Access to technologies that suit individual needs (Domain 5)
  Have access to technologies that are adaptable to the specific needs and preferences of the users. This includes responsive features of both technologies and the healthcare system (including carers) as well as adaptation of devices and interfaces to be used by people with physical and mental disabilities.
3. Experience using technologies
  Feel that using technologies is beneficial (Domain 6)
  Feel that engaging in the use of technologies will help them to manage their health more effectively than by other means.
  Feel in control and secure when using technologies (Domain 7)
  Feel that you have the ownership of personal data stored in the systems and that the data are safe and can be accessed only by people to whom they are relevant (own doctor, own nurse etc.).

### A Framework for Design: Extending the User-Task-Context Matrix With eHealth Literacy

**Figure 1:**
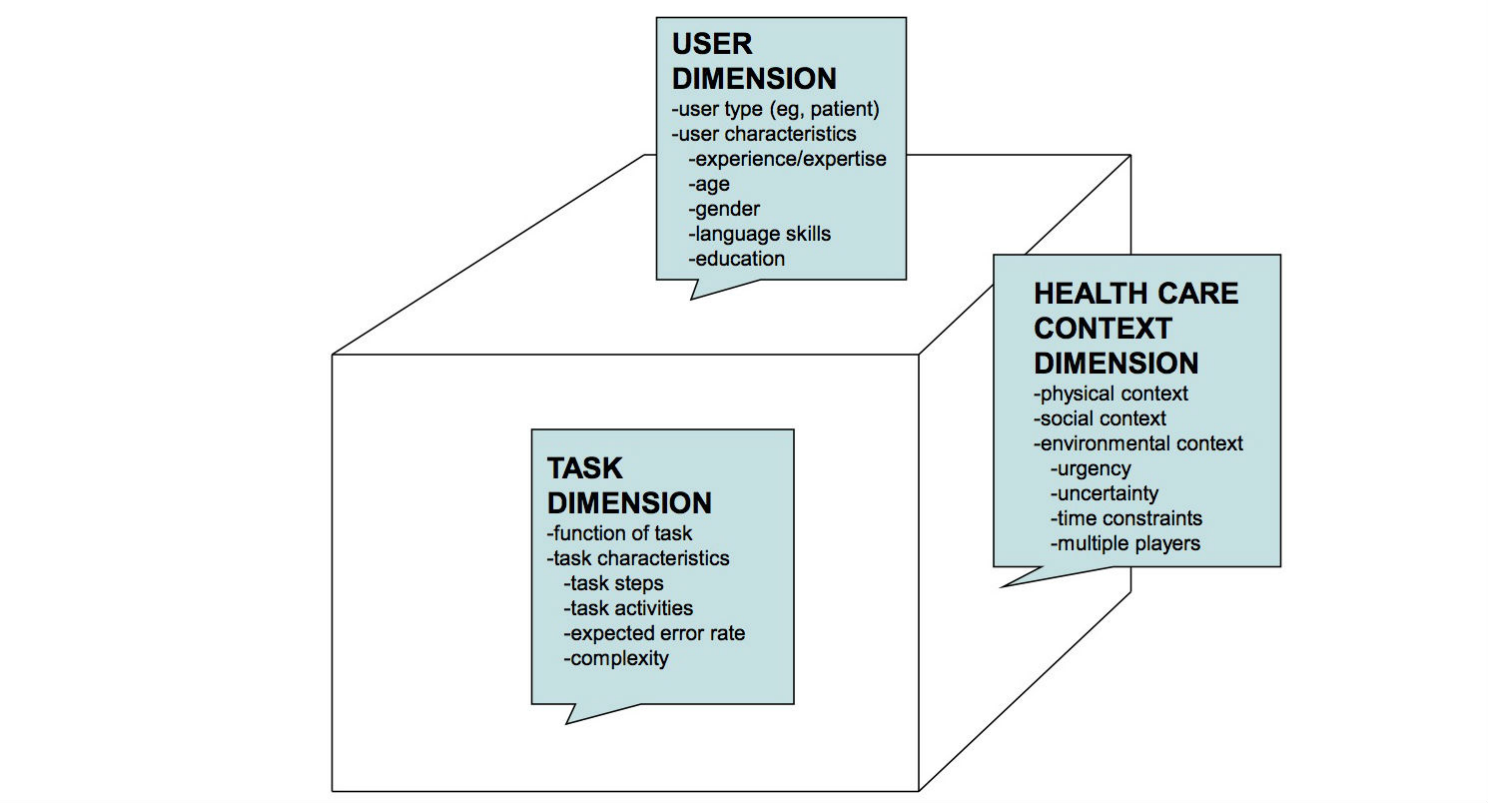
User-task-context matrix for health information technology systems design (adapted from Kushniruk and Turner [23]).

Kushniruk and Turner [23] proposed a concept of a user-task-context matrix to help guide health IT system designers and developers regarding the gathering of user requirements, selection of end users for participatory design as well as for testing and evaluating resultant health IT systems (Figure 1).

Building on the conventional user-task matrix, a standard framework used in development of IT systems for modeling users and the tasks they are expected to be able to carry out using a new technology was developed previously [24]. Kushniruk and Turner [23] proposed that to adequately address safety and quality concerns in health IT, a third dimension was required: the context of use of a health IT system. The original two-dimensional user-task matrix has been successfully applied in IT design and has been described in detail by Hackos and Redish [24] and is part of the human-computer interaction literature. However, in health care settings, in addition to consideration of the user and tasks, the context of use has a significant impact on whether a particular class of user can carry out specific tasks successfully or not using a particular device or information system. “Context” refers to the setting or conditions under which the technology is used; for example, is a system used under urgent or nonurgent conditions? Or is it used in a clinical setting, at home, at work, or while traveling? The “class of user” refers to the generic type of users of an eHealth application, that is, users who share basic characteristics (eg, users of a particular age group or patient users having a specific disease) [23,24]. While designing eHealth applications it is important to have adequate knowledge about all the major types (or classes) of users to target and tailor the eHealth application to meet the needs of each class of users (as different classes of users will have different information needs, understanding of health, and information-processing capabilities). The work of Kushniruk and Turner [23] extends and expands the concept of the user-task matrix to consider the key aspect of context of use. The user-task-context framework has also proven useful in the modeling of user requirements for different health care applications, mostly involving development of systems for health professionals [25,26].

In the context of the development of IT and applications for patients and other health consumers, it is especially important to add the concept of eHealth literacy to the user-task-context matrix in order to create an expanded framework. However, the existing concept of eHealth literacy proposed by Norman and Skinner [5] and subsequently elaborated by Chan and Kaufman [27] does *not* cover the consumer competences required for end users to benefit from and/or have the ability to interact meaningfully with contemporary eHealth systems.

However, with our new modern concept of eHealth literacy, it is now possible to integrate eHealth literacy and the user’s competence into the user-task-context matrix, which makes it possible to generate a framework for health IT design that captures a diversity of consumer-related aspects.

**Figure 2:**
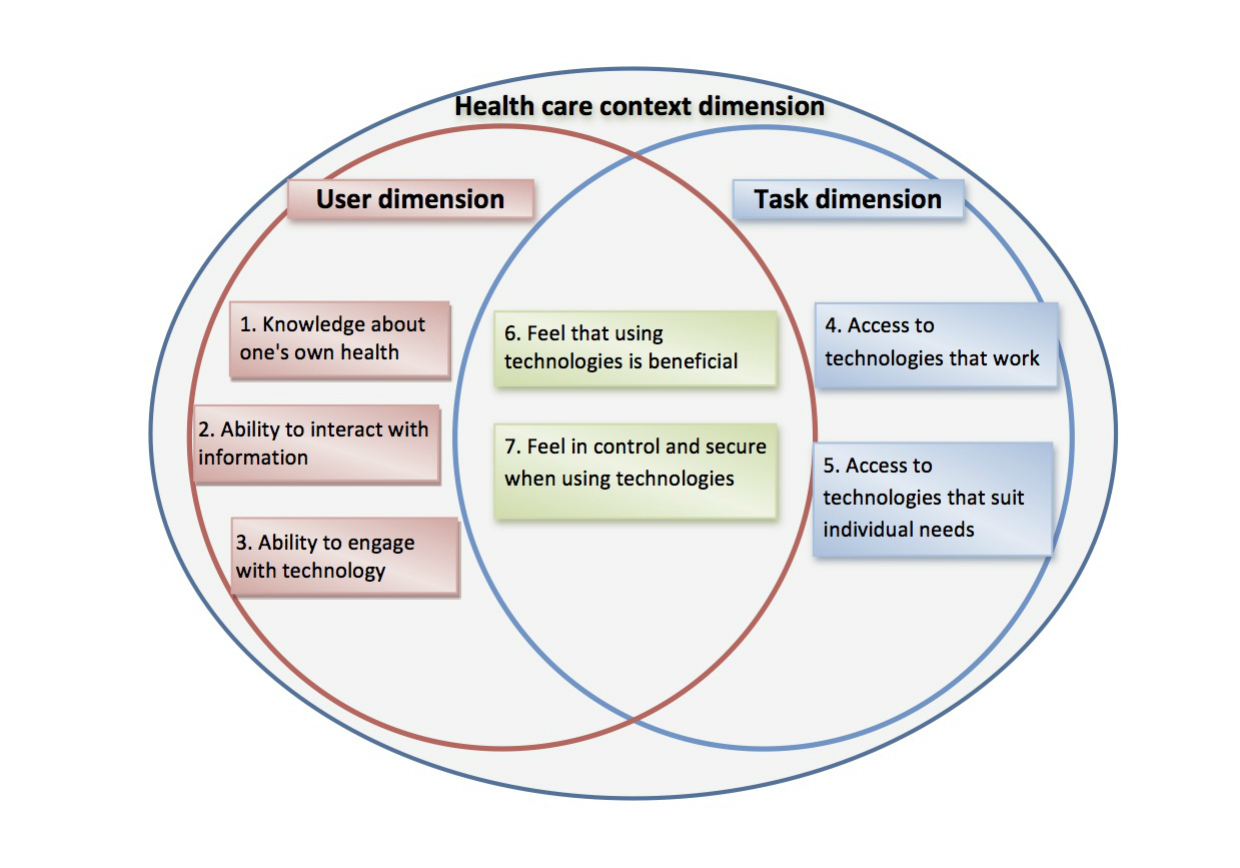
Framework for Design: Expanded User-task-context matrix incorporating eHealth literacy.

The original empirical work that led to this new redefined eHealth literacy concept was intended to highlight to a broad group of potential stakeholders (policymakers, health care providers, clinicians, system designers) the diversity of end-user capability, access, and experience with IT systems. However, further analysis of the concept with regard to the user-task-context matrix revealed that it is possible to extend this matrix to produce a design framework that would enhance consideration of end users in consumer health IT design processes (Figure 2).

Figure 2 illustrates how the seven domains of the eHealth literacy concept relate to the user-task-context matrix model to generate a framework for design. User interactions with digital services always take place in a context of use, illustrated by the large outer circle. The use of digital services is mediated through tasks to be performed and these must be encoded by developers into health IT systems. The services of these health IT systems must, in turn, be intelligible to end users who require the knowledge and skills to find, understand, interpret, and decide how to respond to these outputs (domains 1, 2, and 3). More specifically, the framework highlights that domains 6 and 7 (in the intersection between users and tasks) rely on a detailed understanding of the factors that designers can use to motivate end users and enforce their behavior in the direction of being more self-directed, and domains 4 and 5 require designers to focus primarily on systems and tasks to ensure they can accommodate and support a variety of types of user access and diverse users’ needs. Based on this expanded framework, it is proposed that the concept of eHealth literacy adds value to health IT design processes in at least three ways:

- During requirements gathering, an approach underpinned by principles can be applied to propose, discuss, and test assumptions about the characteristics and capabilities of end users of eHealth systems and applications.
- During participatory design, the selection of participants (users) can take into account the level of eHealth literacy possessed by a broad range of users of the proposed system.
- The potential range of users, defined in terms of eHealth literacy capability, is considered explicitly when modeling user requirements and/or more specifically envisioning classes of end users of the system or application.

Although it is acknowledged that eHealth literacy *is not* the only important dimension or factor that needs to be considered in the development of effective consumer-focused health IT systems and applications, we argue that it is foundational and a critical success factor and that *not* considering end-user eHealth literacy levels may increase safety risks and/or risks of poor outcomes. Other factors in design are consideration of user-specific information architectures and navigational issues, with a growing body of literature addressing the options regarding choices for how to best display information to eHealth consumers and development of guidelines for creating eHealth applications; however, this is out of scope of this paper. The next section illustrates how the framework can be utilized during requirements gathering for the development of consumer-focused eHealth systems and/or applications.

## Results

### An Iterative Five-Step Framework for Understanding Users’ Needs

#### Overview

The aforementioned description illustrates that it is possible to integrate the new eHealth literacy concept (and its 7 domains) with an expanded user-task-context matrix to describe and qualify user requirements and understanding. This section presents the resulting framework that has been produced and structured as a five-step process aimed at supporting eHealth system designers to improve their understanding and more accurately address end-users’ needs, capabilities, and contexts of use to improve the effectiveness and broader applicability of consumer-focused eHealth systems and applications.

**Figure 3:**
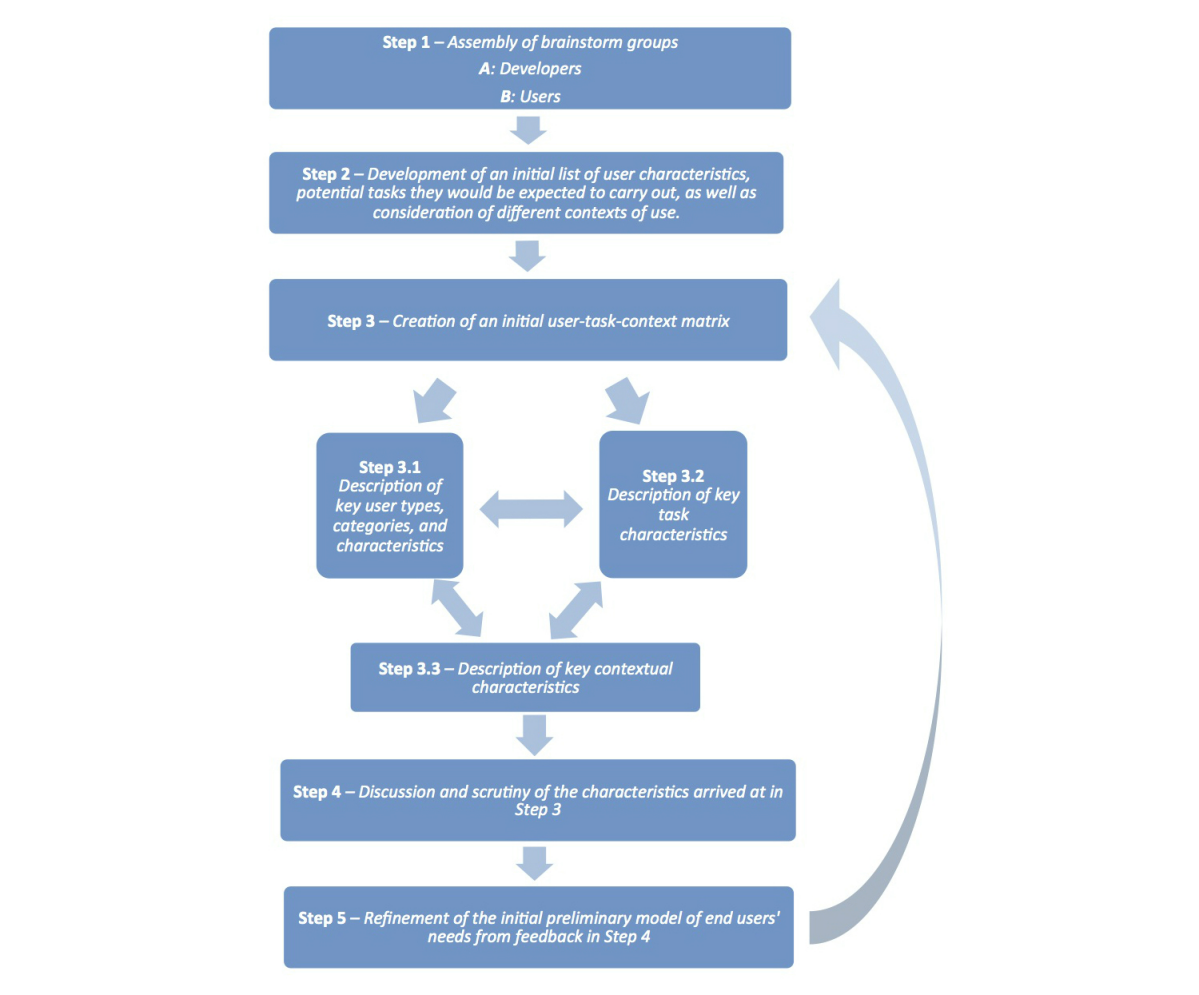
Information technology requirements gathering flowchart.

The following description presents the framework and each of the five steps within it. It highlights that within the framework, generic requirements-gathering approach can be used to enhance understanding, modeling, and reasoning about end-user profiles. Providing designers with personas and/or vignettes can enable them to visualize and understand the particular needs of the span of users. Similarly, specification of requirements can fit into current innovation models such as participatory design [28], rapid contextual design [29], or the biodesign innovation process [30]. The approach can also be recommended when known technologies are planned to be applied in new settings to ensure that the technology can meet users’ capabilities and expectations. The iterative five-step framework includes and expands on the basic steps described by Hackos and Redish [24] for developing a user-task matrix: specifically starting with brainstorming a list of users, creating an initial matrix, and then testing assumptions about users. In the following description, these steps are elaborated to include the consideration of a third dimension—that of context, and to also explicitly highlight consideration of eHealth literacy within the framework when modeling users of health technologies. The steps involved are illustrated in Figure 3 and are described as follows:

#### Step 1: Assembly of a Brainstorm Group

##### Overview

This step consists of two processes. The first involves constituting a group of professionals capable of leading the whole process of requirements gathering. The size of this group should be approximately 8 persons as this will be easier to create a team with trust among the participants. The second involves recruitment of a reference panel.

##### Developers

An initial group with competences within the area for which the requirements are built is assembled. This group should consist of people trained in health informatics, computer science, health care, and behavioral sciences. This group will lead the process including recruitment of a reference panel consisting of end users.

##### User Panel

Recruitment of a reference panel. This panel is most effective when the members are typical end users (laypeople), rather than informed and engaged patients, such as those who may already be active members of patient organizations. The panel will be used in Steps 3.3 and 5 and ideally would consist of 50-150 persons with very wide eHealth literacy levels. However, it should be noted that practical constraints may limit the number of available participants to a smaller number.

#### Step 2: Development of an Initial List of User Characteristics, Potential Tasks They Would Be Expected to Carry Out, as well as Consideration of Different Contexts of Use

For each user, the profile, role, and rights are described. A role can be doctor, nurse, patient, relative, citizen, for example.

In a second iteration, a similar approach can be used with respect to the rights in the system (eg, administrator, superuser, user, supporter). However, in most systems and applications in health care, the professional identity is more important than the rights because consumers, patients, and citizens will expect more rights, and the ability to grant permission to other actors, such as their health professionals and relatives.

In a third iteration, these initial profiles should be aligned with the tasks to be solved and the context (eg, how should the individual profiles act together as collaborators or providers and receivers of services). A key aspect of Step 2 is the preliminary identification of the different categories of end users of the system or application and development of user profiles for each of these categories.

#### Step 3: Creation of an Initial User-Task-Context Matrix

##### Overview

Preliminary ideas from Step 2 are formalized across the user-task-context matrix formalism (as shown in Figure 1). As there are three main dimensions, this step involves three steps (Steps 3.1, 3.2, and 3.3).

Steps 3.1 and 3.2 provide a description of how the associated domains of eHealth literacy are used to qualify the understanding of needs and how a requirement specification is produced, respectively. In Step 3.3, an evaluation of the output from Steps 3.1 and 3.2 is considered in relation to the overarching framework.

It is important to note that the seven eHealth literacy domains together describe the requirements for a successful solution to be offered to users with a high digital confidence (ie, knowledge, skills, and motivation in the context of health). By contrast, the solution will also be beneficial for a wider audience if, for each domain, it is considered regarding how the system can adapt to and handle users with lower knowledge levels, skills, and motivation, by taking design, functionality, and assistive technologies or support tools into consideration.

For this purpose, the seven eHealth domains are qualified for use in the three areas by designing personas with high and low eHealth literacy, respectively, within each of the domains. These 14 descriptors are used in the relevant sections to ensure that the developer team can envisage the typical user span, to ensure the system or application will meet the full range of users.

##### Step 3.1: Description of Key User Types, Categories, and Characteristics

###### Capabilities

####### Knowledge About One's Own Health

Need to consider how users can be supported if they do not know the terms or functions related to their health, how IT systems ensure that the users do not misinterpret information, and how users can be supported to learn more about the specific health area that the system or program intends to cover.

Do links to resources about health and the health care system clearly explain key features in relation to the tasks? What kinds of resources are needed to involve users with low abilities in this area?

####### Ability to Interact With Information

Need to consider how any system addresses the needs of people with low ability to read or calculate or orientate themselves within complex designs. How can the solution assist dyslectics by voice or video and how can figures and calculations be represented in a way that people with dyscalculia can handle?

####### Ability to Engage With Technology

The key questions in this step include how the user interface can address users with different knowledge of how to access systems and interact with them.

The log-in procedures should be simple, the system design should be directed toward integration with other systems, which the typical users are expected to use. The interface should be familiar to other systems and be designed according to international recommendations, standards, and the look of the most used platforms such as Windows and Mac operating systems. The tasks or system should also be easily accessible using various devices and technologies.

##### Step 3.2: Description of Key Task Characteristics

###### Overview

Describe the tasks that different categories of users are expected to carry out through interaction with the system or application being developed. For example, if a task requires input of numeric data, this may impose additional requirement demands on the user being low in domain 2, whereas a task that requires inputs from quick response (QR) codes will not impose numerical challenges. Another example is input or access to personal health data, and how this might influence whether or not the user feels safe and in control with the system. A relevant question is, “Can the task be designed to meet the users’ capabilities or should alternative tasks be created?”

###### Access to Technologies

####### Access to Technologies That Work

How does the system or application document its stability and uptime. After how long a period do the users experience that the system is not accessible? What is the critical uptime to work sufficiently and what is the perspective of the various user groups on access availability?

####### Access to Technologies That Suit Individual Needs

The key characteristics are the usability of the system, adaptability to users with particular needs, and opportunities to collaborate with others if needed.

The interface should be user friendly and designed to be configurable and adaptable to how it is used. Individuals with particular needs due to disabilities should be addressed to avoid inequity in access to the system.

Assistance as built-in support and/or possibilities of getting help from friends, relatives, peers, or health professionals should be considered in the design.

Personas with various disabilities should be created with a focus on the kinds of users who are intended to use the system and which types of disabilities might be expected. Which kinds of individual needs will the typical users have with respect to accessibility, mobility, and variability of interfaces? To what extent will the system need to tailor to individuals with a wide range of individuals’ needs or disabilities?

Next, the tasks are described from the end-user experience perspective.

###### Experience Using Technologies

####### Feel That Using Technologies Is Beneficial

How might the system or application be experienced as beneficial compared with known solutions or other digital products? How can experiences during the use of the solution induce motivation in users with low motivation; for example, through gaming or experienced benefits or victories? How can users with low belief of a new product or accustomed to current solutions be met and taken care of by the solution?

####### Feel in Control and Secure When Using Technologies

How can the users know who has access? How can the interface promote confidence in the system’s security to ensure that people accessing personal data are only those granted permission by the user or, if not, it is clear to the user how and when others access their data? The system should aspire to meet the needs of users ranging from those with a high degree of confidence to those who are concerned about this topic. It should be considered how data management can be transparent and how data are used for users with low confidence or a high degree of mistrust.

##### Step 3.3: Description of Key Contextual Characteristics

As described earlier, the issue of context is critical for developing health care applications that are likely to be used effectively and adopted by end users.

In this step, contexts of use should be identified based on observations in real-life environments and interviews with users and compared with the outputs from Steps 3.1 and 3.2, and should be qualified with respect to identified contextual issues.

The field for observations as well as the users should be identified in Step 1 (subcategory Users) and the users should be recruited in a way that ensures a broad representation of eHealth literacies with variations across domains. The variances can be identified based on semistructured interviews containing questions relating to the descriptors of the domains. It is expected that questionnaires to be applied will be available by 2016. The number of users needed for a panel will vary depending on resources for the development, but the number should be representative for at least one being high and one being low in each of the domains. This can be accomplished by selecting 14 individuals representing the 14 personas described earlier with respect to either high or low level within each of the seven ehealth domains. It will need 128 (2^7^) if a full matrix of variations should be sampled. However, it will probably require testing of a considerably larger population. Therefore, it is recommended to focus on profiles that appear to be the most common within a sample of users. This sample is ideally recommended to be within 50-300 depending on resources (although practical constraints may limit the number to the lower recommended number). For the observed typical combinations of user and task, narratives or fleshed-out use cases should be developed to better define user capabilities in terms of expected tasks. These use cases become an expression of user requirements for systems being developed. They can also be used upon completion of working or partially working prototype versions of the system for setting user- and usability-testing scenarios.

In developing applications and systems for health care professionals, this has typically involved understanding of whether there are contextual issues that would render a system unusable under certain conditions (eg, are there environments or settings where a system would not work?), while other conditions might be seen as being conducive for use of the system. For example, a speech recognition interface to a health information system may work well in a private office, but not at all in the context of a noisy clinical environment. With regard to eHealth applications and systems targeted at patients, laypeople, and the general population, the contextual issue becomes magnified, with an even greater range of settings, situations, and contexts in which a system might be used. To fully understand requirements for a new system or application, an upfront understanding of such contextual facilitators or inhibitors of a system or application is needed.

#### Step 4: Discussion and Scrutiny of the Characteristics Arrived at in Step 3

In this step, the model of users and their interactions with the system in terms of tasks and contextual factors is discussed and scrutinized by the development team. As this will represent only an initial or preliminary model of end users, plans for studying the assumptions and testing them empirically using the “user panel” from Step 1 have to be made. In this step, each of the seven eHealth literacy domains should be taken into account by focusing on how the system meets and fulfills the needs of personas, described in Steps 3.1-3.3. The most important issue here is that the developers undertake the iterations from Steps 3.1-3.3 and ensure that the choices made here remain consistent with the planned requirements for the solution and its user interface, functions, and assistive technologies.

At this stage of development, the designers and developers will test prototypes presenting the content and various ways to interact with it. Here it is important to involve the wide range of identified personas. This may involve discussions with end users, application of contextual enquiry, and usability testing.

#### Step 5: Refinement of the Initial Preliminary Model of End-Users’ Needs from Feedback in Step 4

The model of the end-users’ need developed and tested should be considered in light of feedback from Step 4 by testing the model’s main assumptions. Although developers may hesitate to spend the time and effort needed to refine the initial model represented by the user-task-context matrix, leaving this step out will increase the likelihood that the system or application ultimately developed will not meet the needs of the end users. The refined model can then be used to drive further requirements gathering and streamline the development of system use cases during the design phase and later on during the implementation and testing phases. The model can also be used to create test scenarios.

### A Case Story

As an example of how detailed knowledge about individuals, including eHealth literacy, and the specific context can populate the framework, we briefly present a patient case and describe how considerations related to the seven eHealth literacy domains can feed into Steps 3.1 and 3.2 of the framework. The case was constructed from characteristics identified from a number of real patients in the Epital project [31-33] and author LK’s clinical experience.

The Epital example is chosen as this project consists of a redesign of health services using a whole system approach, service transformation, user centeredness, and especially digital support for both the enrolled people with chronic obstructive pulmonary disease (COPD) and the health care providers.

Angela is a 72-year-old woman with COPD that was diagnosed 5 years ago. Although she takes her medications regularly, she does have exacerbations about 4 times a year and is in need of hospitalization or referral to an emergency room 2 times a year. Because of the condition, she has anxiety, is slightly cognitively impaired, especially when exacerbations are emerging, and it is at this particular time she needs to use her Epital navigator to connect to her digital service and trust that it works instantly. Owing to treatment of her COPD with beta-2-adrenergic stimulators, she suffers from tremor of her hands, which is exaggerated when her condition worsens, partly due to an increased intake of medicine and partly due to an increase in anxiety. She has an average score in self-reported health literacy, only few errors in a test of health literacy primarily related to calculations of medicine doses, whereas her understanding of measurements related to her condition is found to be insufficient and makes it difficult for her to interpret and act on data outside the normal range. Although she scores on average in most tests, the confidence and speed of performance are not good.

Table 1 describes how this profile can be utilized when designing a home monitoring solution for patients with COPD.

**Table 1:** Using the seven-domain eHealth concept in Health information technology design.

eHealth literacy domains	Description
Knowledge about one’s own health (Domain 1)	The patient has sufficient basic information but her understanding of more complex data makes it difficult to interpret her own measurements. Here it should be considered to build in a function that assists interpretation.
Ability to interact with information (Domain 2)	Angela is able to read but has some problems with calculation, which may be taken into consideration here.
Ability to engage with technology (Domain 3)	She scores average in confidence and skills with computers, and thus, the solution should either include training for the particular system or assisting functions should be included, which will address the areas in which the patient is insecure and has a low digital competence.
Access to technologies that work (Domain 4)	Her condition could, in a short time, be life threatening and it is therefore important to allow for feedback about connectivity and a way to secure backups to avoid an increase of her anxiety, which is often related to a lack of confidence in health information technology services.
Access to technologies that suit individual needs (Domain 5)	She has tremor of her hands and needs a very simple button or a technology like pointers to be sure that she presses the right areas. She will also need a solution that provides interaction with real persons when her exacerbations are severe because she will not be able to interact with the technology without voice or video support.
Feel that using technologies is beneficial (Domain 6)	Angela has been introduced to a home monitoring device in the form of a tablet computer. It should be considered how a simple user interface can be developed because she has felt insecure when she performs tasks indicating uncertainty in relation to the technology and her own condition. This may be influenced by her medicine and possible anxiety state.
Feel in control and secure when using technologies (Domain 7)	She has scored as being confident about the Internet and the use of computers, and therefore, this domain may not be of concern.

## Discussion

The proposed expanded framework has been designed to provide a user-friendly framework for design. It will directly assist health systems policy development and decision making because it can be deployed to ensure that the design process takes the users’ capabilities, and the circumstances in which resulting products are to be used, into consideration. This will maximize the probability of generating usable, safe, and highly implementable IT applications and systems, and generate the intended positive health outcomes. From previous work, it has been found that fitting health information system design to users’ understanding and capabilities leads to a thoughtful design and more usable and useful systems and that ease of use and usefulness are also associated with not just more effective systems, but also with safer systems [2]. It should be noted that the approach described in this paper gives an overall framework for considering users of health information technologies to highlight consideration of eHealth literacy explicitly. As such, it will complement specific data-collection methods (eg, the use of the think-aloud method or simulations) used in obtaining detailed information about users, their needs, and their capabilities [26]. In addition, the approach can be applied not only for design, but also for documentation of eHealth application requirements that can be consulted once an application is developed to help target end-user support (eg, by assessing whether individuals of differing user classes can do the tasks the eHealth application was designed to support once in widespread use). It should also be noted that the approach could be applied in the development of eHealth applications targeted for individuals as well as for groups. For example, in development of regional or national personal health record and personal health portals, consideration is needed of what the major classes of users of the application are, their eHealth literacy, and the type of tasks they would find useful (including collaborative and socially oriented tasks) to perform using the application. As there is a move toward greater use of social media this need will only increase.

The proposed new concept of eHealth literacy [34] has similarities to Norman and Skinner’s model with six core literacies [10] within the areas of tradition alliteracy, health literacy, computer literacy, information literacy, similar to the domains “1. Knowledge about one’s own health,” “2. Ability to interact with information,” and “3. Ability to engage with technology.” The media and science literacy are not represented as strongly as in our new concept. By contrast, based on the statements in the development process, we have got a unique insight into how the users (ie, patients, health professionals, and technology developers) are thinking. These insights can be used as a framework to describe where developers need to be aware of in the design processes as it is presented in this paper.

Thus, we are able to present an expanded model of the user-task-context matrix, which qualifies needs to be addressed not only with respect to the users, but also how they expect tasks and systems to be designed and also how the intersection between the users’ competencies and the service design should be addressed to motivate the users, having them feel safely in control, and being able to interact. In this way, our concept with its underlying statements contributes to a new understanding of eHealth literacy. The concept also includes subjects previously identified to be essential to ensure usage of health technology [35,36], that is, trust that is embedded in the domains “4. Access to technologies that work” and “7. Feel in control and secure when using technologies and interpersonal relationships,” which is embedded in the domain “5. Access to technologies that suit individual needs” where statements from the development process cover aspects of being able to share data with relatives, support relatives, or receive support from others. This aspect is also shortly described in our recommendations in Step 3.2. We find that our seven domain eHealth literacy concepts covers both the important domains of the definition and the model by Norman and Skinner. At the same time, it also covers other dimensions of importance for understanding the interaction between users and the digital services offered by health care providers in the future.

This new eHealth literacy concept has turned out to have similar focus on several areas of the user-task-context matrix suggested by Kushniruk and Turner [23]. Because this matrix lacked dimensions for user characteristics related to eHealth literacy, we have integrated the concept of eHealth literacy and the matrix into an expanded framework, which can be used to produce requirements for designers. It is anticipated that the framework presented directly supports the more accurate identification of differences in users, and their contexts of use, and how these factors interact with usability and the risk of unintended consequences from health IT systems. The new concept of eHealth literacy and the expanded framework presented suggest that there are ways for opening up new conversations about innovative ways of thinking, designing, and empowering health consumers in their use of health information and health information systems. The expanded framework contains personas and vignettes/narratives, which illustrate the needs of users with both high and low competences.

To maximize the impact of the framework, we have specified its application within a structured process that involves a multidisciplinary small group to facilitate and lead the process and to create a user panel of laypeople to ensure connection to the real world. In this way, the presented framework can expand how users are involved in the initial phases of other innovation models such as the participatory design [28] or rapid contextual design [29]. The proposed process systematically qualifies them through a grounded understanding of the mandatory areas that must be understood and anticipated to maximize usability from both an end-user and a system approach. The framework will also be of great importance when the Stanford Biodesign process is used in development of eHealth solutions. The first step of the framework will be skipped at this point in the Biodesign process because cross-disciplinary teams have already been established. Steps 2-5 can be embedded in the invention phase when prototyping begins to ensure that the involved stakeholders and patient segments are understood and addressed with respect to eHealth literacy, and that specific criteria are included in the need statements to ensure a safe and usable product.

Although there is extensive evidence that health literacy is associated with health outcomes [37], and that eHealth interventions may improve health outcomes [38], there is limited evidence connecting eHealth literacy and the use of eHealth interventions. One reason for this is that in many cases projects are not designed on a large scale and may not disseminate knowledge based on evidence [39].

Before the full benefit of the framework can be obtained, methods to estimate individuals’ eHealth literacy are required. At present, we are developing such tools, which are expected to be available by the end of 2015. At this point, it is recommended to use semistructured interviews based on the descriptors in Textbox 1 to classify users according to their eHealth literacy.

For developers, it is expected that the proposed expanded framework for design will ensure that future health IT products involving innovation, design, and maturation phases will be designed to accommodate the needs of a variety of users from a system-design approach involving eHealth literacy. The intention is to ensure a robust and safe system where the developers involved will also understand the situations and contexts in which the system will be used. Although the entire expanded framework has not yet been implemented in full, we invite designers to use it and to create research projects where its effects can be documented.

Developing effective eHealth technologies requires an understanding of the needs of end users from multiple perspectives. In this paper, we have proposed and detailed a framework for modeling users’ needs for eHealth that merges prior work in development of a user-task-context matrix with the emerging area of eHealth literacy. This framework is intended to be used to guide design of eHealth technologies and to make requirements explicitly related to eHealth literacy, enabling a generation of well-targeted, fit-for-purpose, equitable, and effective products and systems.

## Abbreviations

COPD: chronic obstructive pulmonary disease
eHEALS: eHealth Literacy Scale
IT: information technology
QR: quick response
